# Bibliographical Department

**Published:** 1881-04

**Authors:** 


### BIBLIOGRAPHICAL DEPARTMENT.

"Nothing extenuate, nor set down aught in malice."

THE SCIENTIFIC AMERICAN AND SCIENTIFIC AMERICAN SUPPLEMENT are undoubtedly the most valuable periodicals in the departments of science which they discuss and illustrate, that are published in this country. While the text is always interesting and instructive, the engravings and illustr·tions are elaborate and superb. Published by Munn & Co., N. Y., at $3.20 and $5.00 per annum.

A PRACTICAL TREATISE OF THE SKIN. BY LOUIS A. DUHRING, M. D., *Professor of Diseases of the Skin in the Hospital of the University of Pennsylvania; Dermatologist to the Philadelphia Hospital; Consulting Physician to the Dispensary for Skin Diseases; Author of the "Atlas of Skin Diseases," etc.* Second Edition, Revised and Enlarged, Philadelphia, J. B. Lippincott & Co., 1881.

The foregoing is the title page of one of the most valuable works published upon a specialty in this country.

Dr. Duhring has performed his task in such a practical common sense manner, and shown himself such a complete master of the subject, as to interest any one at once in what he writes; and there are very few, if any of us, who will not experience a feeling of pride on perusing the book, that it was written by one of our own countrymen.

The author has made much improvement in the work since the first edition was published, five years ago, and it is now a most valuable practical treatise on dermatology; probably the very best in our language.

The publishers have executed their part of the work in an admirable manner.

*Lectures Upon Diseases of the Rectum and the Surgery of the Lower Bowel, Delivered at Bellevue Hospital Medical College, By W. H. Van Buren, M. D., LL. D. (Yalen), Professor of the Principals and Practice of Surgery in the Bellevue Medical College: One of the Consulting Surgeons of the New York Hospital; of the Bellevue Hospital; of the Presbyterian Hospital; Formerly President of the New York Pathological Society, and Vice President of the New York Academy of Medicine; Member of the New York Surgical Society; Corresponding Member of the Surgical Society*

*of Paris. etc., etc. New York, D. Appleton & Co., 1881. Price $3.*

The above announcement of the second edition of Professor Van Buren's valuable book, is just to hand, and a comparison with the previous one shows he has made considerable improvement in its pages. His more enlarged experience and extended research have enabled him to present to the profession a most valuable work on a specialty which was hitherto too much neglected by the regular profession. In fact, the treatment of many diseases of the rectum has been largely in the hands of quacks and charlatans for many years past. The tendency of this book must be to lift this department of surgery out of the hands of adventurers, and to place it upon a level with the other specialties of the day. Dr. Van Buren's reputation as a surgeon is sufficient to attract the attention of the profession to this edition, and it will doubtless soon find a place upon the table of all the earnest surgical workers of the country. The style is flowing and graceful, and the publishers have gotten the book up in their usually neat and readable manner.

IMPERFECT HEARING AND THE HYGIENE OF THE EAR. *Including Nervous Symptoms, etc., etc.. with Home Instruction for the Deaf,* By LAURENCE TURNBULL, M. D., PH. G., *Aural Surgeon Jefferson Hospital; Physician to the Department of Diseases of the Eye and Ear, Howard Hospital, Philadelphia; Fellow of the American Association for the advancement of Science, and Member of the American Medical Association, etc. Third edition, with illustrations.* Philadelphia: J. B. Lippincott & Co., 1881. Probably all of our readers, or at least, those who have been in practice for a decade or more, are familiar with "Turnbull's Tinnitus Aurium," as the former editions were known; but all of them who will possess themselves of the third edition will find the money well invested that is paid for it. The author's opportunities have been ample both at home and in Europe, for working up and bringing the book fully abreast of the times, and he has done so in an able manner. We are indebted to the courtesy of the author for our copy.

We are under obligation to Dr. E. Janssens, Inspecteur du Service de santa de la ville de Bruxelles, etc., for the following interesting Brochures and reports, viz: "Prophylaxie Administrative contra la propagation des maladies contagieuses, et specialement de la variole. De L'Inspection Hygienique et Medicale, dans les Ecoles. Ville de Bruxelles: Annuaire Demographique et Tableaux Statistiques des causes de Deces. And, Rapport sur les operations du Bureau D'hygiene et sur la salubrite Publique de la ville de Bruxelles, 1879.

STRANGULATED VEINS OF THE UTERUS, *and other papers Gynæcological and Surgical*, By THOMAS H. BUCKLER, M. D. of Baltimore. Cambridge: Printed by the Riverside press, 1881. All the papers contained in this Brochure are interesting, and some of them of much practical value.

UNIAS MEDICA.—Publicacao Mensal. Redactores Dr. Julio de Moura. Dr. Moneorvo, Dr. Moura Brazil. Dr. Silva Aracy's. No. 1—Janeiro de 1881, Rio de Janeiro, Brazil.

MEDICAL TIMES AND GAZETTE.—A weekly Journal of Medicine, Surgery and the Collateral Sciences. New York, March, 1881.

THE HOMŒOPATHIC TIMES.—A Monthly Journal of Medicine, Surgery and the Collateral Sciences. New York, March, 1881.

All the above journals have been received and placed upon the exchange list.

JOHNS HOPKINS UNIVERSITY CIRCULAR, NO. 9, For March. Published with the approbation of the Board of Trustees, has been received, and will be referred to in next issue.

We have received through the courtesy of Dr. H. P. C. Wilson, the *Circular of St. Vincent's Hospital, Baltimore.* The institution is under the charge of the Sisters of Charity.

PACIFIC MEDICAL AND SURGICAL JOURNAL. HENRY GIBBONS, M. D., and HENRY GIBBONS, JR., M. D., Editors and Proprietors, March, 1881.

THE DETROIT LANCET, A Monthly Exponent of Rational Medicine, Edited by LEARTUS CONNOR, A. M., M. D. We take pleasure in adding these two old and well conducted journals to our list of exchanges.

THE NORTH AMERICAN REVIEW for May contains a striking article by the Hon. David Dudley Field on "Centralization in the Federal Government." That our polity is rapidly advancing in the direction of centralization is demonstrated by the author; but whether centralization is really a formidable evil or only a bugbear is a question which men will probably continue to decide according to their several political predilections. Whatever the reader's bias, Mr. Field's paper will command his respectful attention, and it will be read with interest and profit. The second article is upon the new revision of the Bible, by the Rev. Dr. Schaff, of the American Committee of Revision. Mr. Justice Strong writes of "The Needs of the Supreme Court," and advocates the establishment of a court of appeals, intermediate between the U. S. Supreme Court and the circuit courts. The Hon. George Q. Cannon, the first advisor and the President of the Mormon Church, and delegate to Congress, makes a vigorous defence of "Utah and its people." The question, "Shall Americans build Ships?" is considered by Mr. John Roach, the ship-builder, who brings forward a large number of facts to prove that the people of the United States must build ships if they would hold a place among maritime nations. The other articles are "The Life-Saving Service," by the Hon. S. S. Cox; "The Ruins of Central America," by M. Charney; and finally an attack on evolution philosophy, written in a vein of the finest irony, and entitled "What Morality Have We Left?"

THE PATHOLOGIST, Vol. I, No. 2, Brooklyn, N. Y., and THE JOURNAL OF MATERIA MEDICA, Published by Geo. H. Tilden, New Lebanon, N. Y., have reached us just as this department was ready for the printers; and will be noticed hereafter.

We are indebted to our esteemed friend, Hon. Alex. H. Stevens, for a copy of the addresses on the presentation of the statue of Jacob Collamar of Vermont; delivered in the House of Representatives, February 15th, 1881.

THE SANITARY ENGINEER, is one of the most useful and valuable hygienical publications, in this country. It is published in New York at the low price of $3.00 per annum. Exchange list.

FIRST BIENNIAL REPORT OF THE NORTH CAROLINA BOARD OF HEALTH.—We are indebted to the courtesy of Dr. Thomas F. Wood for a copy of this interesting book, and hope to be able to utilize some of its contents in future issues of the journal.

DR. I. W. SINGLETON, of Paducah, Ky., has kindly sent to us a re-print of "Suggestions on the Management of National Labor," which contains some good and timely suggestions.

EL MEDICO Y CIRUJANO CENTRO-AMERICANO. *Publicacion Mensual de Medicina, Cirugia, Farmacia, Y Los Ramos Colateres. Guatemala, C. A. Febbero de 1881,* has been received and placed upon our exchange list.

THE KANSAS CITY REVIEW OF SCIENCE AND INDUSTRY.—We are happy to receive and place upon our exchange roll, this valuable periodical. It is published at Kansas City, Missouri, and ably edited by Theo. S. Case, Esq. The issue before us is full of useful and interesting reading.

